# Light-induced olefin metathesis

**DOI:** 10.3762/bjoc.6.127

**Published:** 2010-11-23

**Authors:** Yuval Vidavsky, N Gabriel Lemcoff

**Affiliations:** 1 Chemistry Department, Ben-Gurion University of the Negev, Beer-Sheva 84105, Israel

**Keywords:** catalysis, light activation, olefin metathesis, photoactivation, photoinitiation, photoisomerisation, RCM, ROMP, ruthenium, tungsten

## Abstract

Light activation is a most desirable property for catalysis control. Among the many catalytic processes that may be activated by light, olefin metathesis stands out as both academically motivating and practically useful. Starting from early tungsten heterogeneous photoinitiated metathesis, up to modern ruthenium methods based on complex photoisomerisation or indirect photoactivation, this survey of the relevant literature summarises past and present developments in the use of light to expedite olefin ring-closing, ring-opening polymerisation and cross-metathesis reactions.

## Introduction

The metal catalysed olefin metathesis reaction [1–8] has undoubtedly become one of the most widely used methodologies for the formation of carbon–carbon bonds. Its ubiquitous use in polymer chemistry [9–13] and natural product [14–17] and fine chemical synthesis [3,18–20], ultimately led to the 2005 Nobel Prize award to Yves Chauvin, Richard Schrock and Robert Grubbs [21] for the development of this reaction. Since, olefin metathesis has seen much progress, such as the use of new ligands for aqueous applications [22–26], asymmetric synthesis [27–30] and latent catalysis [31]. Among the methods used to activate latent olefin metathesis catalysts we find, chemical methods [32] and physical methods; such as the use of thermal energy [33], mechanochemical energy [34] and, perhaps more conveniently, the use of light [35]. In this review we summarise the early beginnings of light induced olefin metathesis by the use of ill defined tungsten complexes, up to the most recent developments in light induced ruthenium based isomerisation and activation.

## Review

### Early tungsten catalysed photometathesis

The first examples for photoinitiated metathesis were published independently by Dubois and McNelis in 1975 using simple tungsten hexacarbonyl as the metal initiator in carbon tetrachloride solvent [36–37]. This straightforward, cocatalyst free technique encouraged many research groups to investigate and develop this system.

**Scheme 1:**
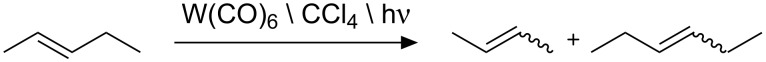
Light activated metathesis of *trans*-2-pentene.

Dubois demonstrated the photoinduced metathesis (335 nm) of *trans*-2-pentene to 2-butene and 3-hexene in 50% conversion; while McNelis used a 254 nm Rayonet reactor for the metathesis of hept-3-ene, pent-2-ene, and *E,E*-deca-2,8-diene (Scheme 1).

**Scheme 2:**
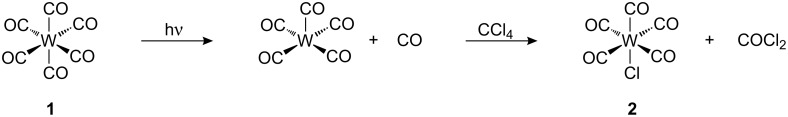
Light induced generation of metathesis active species **2**.

In the Dubois paper, when either light or carbon tetrachloride was excluded, no reaction could be observed. Furthermore, this research led to the proposal of the controversial mechanism shown in Scheme 2, which includes the generation of phosgene and the proposed active species, chloropentacarbonyl tungsten (**2**).

Alternatively, McNelis proposed that the active species was actually dichlorotetracarbonyl tungsten and demonstrated that phosgene was not generated by illumination of **1** when oxygen was excluded [38].

Following these first communications, Krausz, Garnier and Dubois published a series of papers investigating the photoinduced olefin metathesis with complex **1** [39–42]. Their main conclusions were:

*a:* W(CO)_5_ and CO are created by photolysis of W(CO)_6_. The reactivity of W(CO)_5_ was found to be solvent dependent.

*b:* The intensity of irradiation, and the concentrations of both olefin and catalyst had a significant effect on reaction yields.

*c:* The RCH=CCl_2_ produced in the reaction was a result of a reaction between a tungsten dichlorocarbene species and the double bond.

Research on the photo-activation of W(CO)_6_ was further expanded by Harfouch et al. [43–44], Matsuda et al. [45], Szymańska-Buzar and Ziόłkowski [46–47] and Zümreoglu [48], and was first reviewed in 1988 by Szymańska-Buzar [49]. The main conclusions from this wave of research dealt with the mechanistic role of the halide additives, as well as diverse reaction conditions, such as the use of other metals (i.e., Cr and Mo) that usually led to addition type reactions instead of metathesis.

In later work, Mol et al. [50] determined the heterogeneous character of the active catalytic species obtained on irradiation of **1** in CCl_4_, supporting the previous proposal by Harfouch and coworkers. In another example of heterogeneous light induced metathesis, Shelimov and Kazansky [51] also found that silica supported molybdenum trioxide (MoO_3_/SiO_2_) could be activated by UV irradiation under an alkane atmosphere in the metathesis of propene and 1-hexene.

More recently, Sundararajan et al. [52–54], and Higashimura et al. [55] applied W(CO)_6_/CX_4_/hν methodologies for the polymerisation of alkyne derivatives, especially phenylacetylene. These works were based on earlier observations by Katz et al. [56–57] and Geoffrey et al. [58] that acetylenes irradiated in the presence of tungsten complexes form metal carbenes that can produce polymeric species.

### Well-defined tungsten catalysed photometathesis

**Figure 1:**
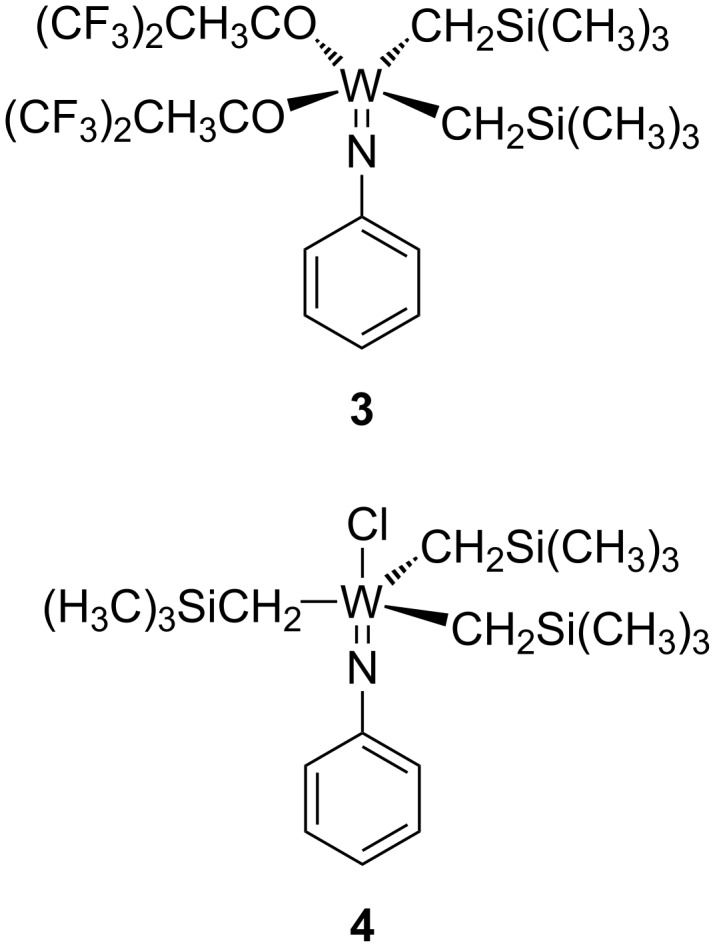
Well-defined tungsten photoactive catalysts.

The first example of well-defined early transition metal complexes for photocatalysed ROMP (PROMP) **3** and **4** (Figure 1) was published by van der Schaaf, Hafner, and Mühlebach [59].

Complexes **3** and **4** displayed reasonable thermal stability in solution (no decomposition was observed after 1 d at 80 °C), but were moisture sensitive and had to be handled under an inert atmosphere. Complex **3** slowly polymerised dicyclopentadiene (DCPD) in the dark at 60 °C, in contrast, when exposed to light even at room temperature, polymerisation was rapid and complete after 15 min. Enhanced behaviour was observed with complex **4**, which boasted true latency [60] and did not show any polymerisation of DCPD at 80 °C. Whilst a solution of **4** and DCPD can stand for days with no apparent polymerisation, irradiation with UV light led to fast polymerisation.

### Ruthenium catalysed photometathesis

**Figure 2:**
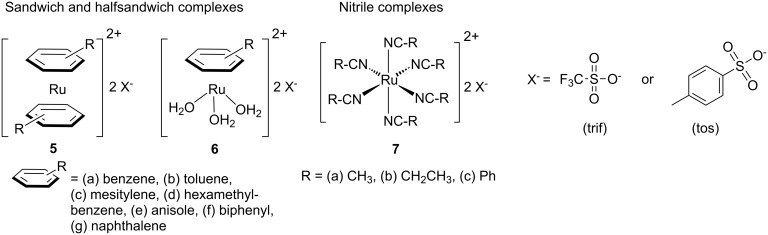
The first ruthenium based complexes for PROMP.

**Figure 3:**
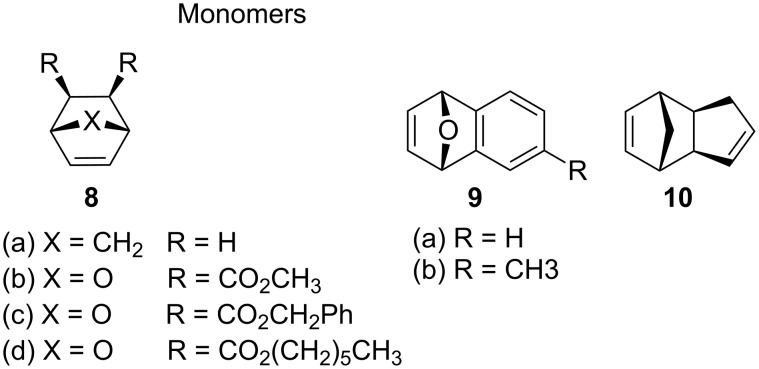
Cyclic strained alkenes for PROMP.

The first example for PROMP using ruthenium initiators was disclosed by Mühlebach et al. in 1995 [61]. Three main types of ruthenium-based precatalysts: η^6^-arene sandwich complexes, half-sandwich complexes and nitrile complexes (Figure 2) were shown to promote the polymerisation of strained cyclic alkenes (Figure 3) when irradiated by UV light.

Advantageously, the systems showed none to moderate activity for normal ROMP, and were not sensitive to oxygen and humidity compared to the tungsten initiators described above. Notably, heating the sandwich catalysts for more than 24 h at 50 °C in the presence of the monomers did not induce polymerisation, on the other hand, the nitrile complexes displayed low activity even at room temperature. UV irradiation at 364 nm significantly enhanced the activity of all these complexes. The activity of the compounds, including comparisons with thermally active catalysts, are summarised in Table 1.

**Table 1:** Activation of ruthenium complexes with and without irradiation^a^.

Entry	Compound	Catalytic activity	
		Thermal	hν^b^

1	[Ru(H_2_O)_6_](tos)_2_	Very high	
2	[Ru(H_2_O)_6_](trif)_2_	Very high	
**Half-sandwich complexes**			
3	[(C_6_H_6_)Ru(H_2_O)_3_](tos)_2_	Medium	High
4	[(toluene)Ru(H_2_O)_3_](tos)_2_	Weak	Medium
5	[(hexamethylbenzene)Ru(H_2_O)_3_](tos)_2_	Very weak	Very weak
6	[(C_6_H_6_)Ru(acetonitrile)_3_](tos)_2_	Medium	
**Sandwich complexes**			
7	[(C_6_H_6_)_2_Ru](tos)_2_	None	High
8	[(C_6_H_6_)Ru(toluene)](tos)_2_	None	Medium
9	[(C_6_H_6_)Ru(mesitylene)](tos)_2_	None	Weak
10	[(C_6_H_6_)Ru(hexamethylbenzene)](tos)_2_	None	Weak
11	[(mesitylene)Ru(hexamethylbenzene)](BF_4_)_2_	None	Weak
12	[(hexamethylbenzene)_2_Ru](tos)_2_	None	Very weak
13	[(C_6_H_6_)Ru(anisole)](tos)_2_	Weak	High
14	[(C_6_H_6_)Ru(biphenyl)](tos)_2_	None	Very high
15	[(C_6_H_6_)Ru(naphthalene)](tos)_2_	Medium	Very high
16	[(C_6_H_6_)Ru(chrysene)](tos)_2_	Medium	Very high
17	[(C_6_H_6_)Ru(tetramethylthiophene)](tos)_2_	None	Very high
18	[(C_6_H_6_)Ru(triphos)](tos)_2_	None	None
19	[(C_6_H_6_)Ru(1,2,4-C_6_H_3_Me_3_)](BF_4_)_2_	None	Weak
20	[(C_6_H_6_)Ru(1*R*,2*S*-trans—C_12_H_16_O)](BF_4_)_2_	None	Weak
21	[(C_6_H_6_)Ru(1*S*,2*R*-trans—C_12_H_16_O)](BF_4_)_2_	None	weak
**Nitrile complexes**			
22	[Ru(acetonitrile)_6_](tos)_2_	Weak	High
23	[Ru(acetonitrile)_6_](trif)_2_	Weak	High
24	[Ru(propionitrile)_6_](tos)_2_	Weak	High
25	[Ru(propionitrile)_6_](trif)_2_	Weak	High
26	[Ru(benzonitrile)_6_](tos)_2_	Weak	Medium
27	[Ru(benzonitrile)_6_](trif)_2_	Weak	Medium

^a^monomers: **8a** or **8b**, concentration 50–200 mg/mL; catalyst, 1 wt %; ^b^irradiation with a Hg lamp for 15 min prior to the addition of monomer.

Overall, the polymers obtained from norbornene and oxanorbornene derivatives had high molecular weights (*M*_w_ > 150 kDa) and also high monomodal polydispersities (*M*_w_/*M*_n_ > 2.3). Polymerisation was also found to occur if the monomer was added after irradiation of the complex. In addition, the initial rate of polymerisation was found to be linearly dependent on the irradiation time. Conversely, irradiation above 420 nm did not initiate polymerisation for most of the complexes investigated.

**Scheme 3:**
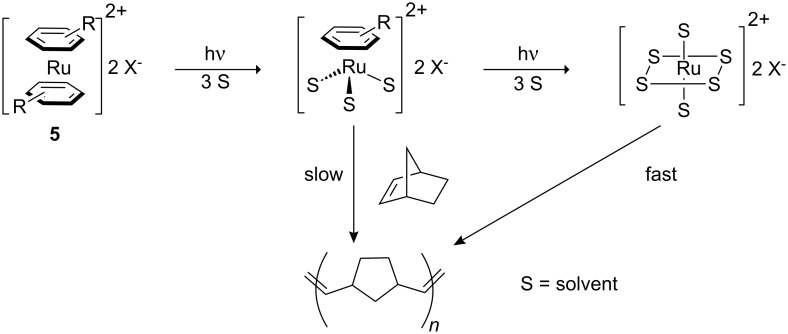
Proposed mechanism for photoactivation of sandwich complexes.

As in most systems of this type, activation of the precatalyst is dependent on the photochemically induced cleavage of a metal–ligand bond which leads to the active species. The mechanism for initiation of the sandwich compounds was proposed to proceed through gradual photodissociation of the arene ligand, followed by solvation of the photochemically excited molecule (Scheme 3).

Both the intermediate half-sandwich species [(η^6^-arene)Ru(solvent)_3_]^2+^ and the fully solvated complex [Ru(solvent)_6_]^2+^ are thermally active ROMP catalysts for strained bicyclic olefins, the latter being the more active. Thus, Mühlebach concluded that PROMP results mainly from the phototransformation of the sandwich complex to the fully solvated complex. The nitrile complexes are proposed to be activated by a similar mechanism where several species of the type [Ru(RCN)_6−x_(H_2_O)_x_] are responsible for the polymerisation initiation.

Disadvantages of these catalysts are the overall moderate activities achieved and that they are only soluble in polar solvents, due to their cationic character. On the other hand, the complexes are readily available and the use of aqueous solvents as the reaction media can also be envisaged as an attractive feature.

**Figure 4:**
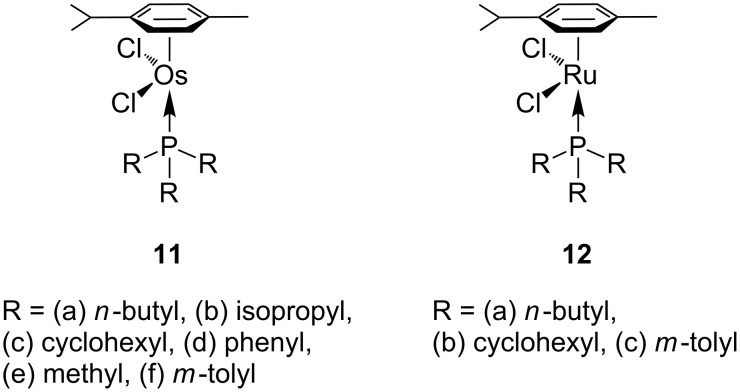
Ruthenium and osmium complexes with *p*-cymene and phosphane ligands for PROMP.

Mühlebach, Hafner and van der Schaaf [62] carried on the development of ruthenium and osmium photoactivated catalysts (Figure 4) by adding a bulky phosphane ligand to the complex. Thus, a more active and soluble neutral species with the anionic ligands bound to the metal could be obtained. The same concept of arene displacement by UV radiation was used for the release of a *p*-cymene ligand to produce a more reactive catalytic species.

Osmium precatalysts **11a**–**f** did not polymerise norbornene under standard conditions. However, 5 min irradiation of a toluene solution of the complex with a 200 W Hg lamp led to active catalysts. The more active catalysts were those possessing more sterically hindered phosphane ligands. Thus, complexes with larger cone angles θ, such as **11b** (θ = 160°) and **11c** (θ = 170°) showed strong metathetic activity for PROMP of norbornene and dicylopentadiene in toluene solution or even in aqueous dispersions; by contrast, complexes **11a**,**d**,**e**,**f** (θ = 130°, 145°, 120°, 150°) showed slow no reaction even after UV irradiation. The ruthenium complexes **12** showed much higher reactivity in the polymerisation of norbornene, albeit none of these complexes was completely thermally latent for this reaction. The remarkable tolerance of **12b** to impurities and water, was highlighted by the fact that polymerisation can take place in water dispersions containing fillers such as SiO_2,_ AI(OH)_3_, or CaCO_3_ up to a loading of 70 wt %. The electrical and mechanical properties of PDCPD were preserved, making this system highly interesting for novel applications. The work of Mühlebach, Hafner and van der Schaaf on photoinduced ring-opening metathesis polymerisation was reviewed in 1997 [63].

**Figure 5:**
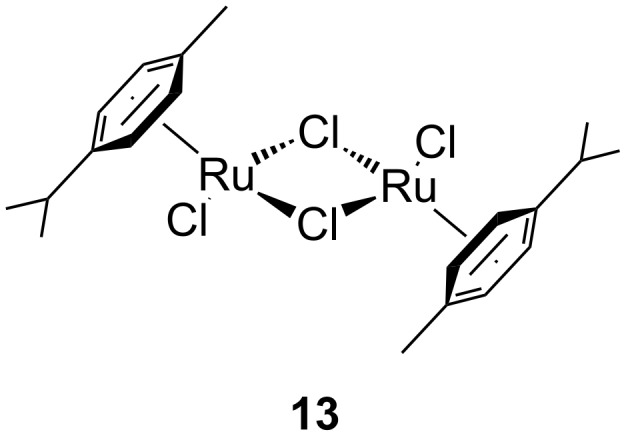
Commercially available photoactive ruthenium precatalyst.

The interest in photoactivated olefin metathesis motivated Fürstner [64] to use complex **12b** for photoinduced ring closing metathesis (RCM) by using regular neon light or strong daylight as a photon source instead of the UV lamps used thus far. Indeed, **12b** catalysed the ring-closing metathesis reaction of diallyl tosylamide in 90% yield when illuminated by neon light. Alternatively, the commercially available dimer complex **13** (Figure 5) mixed with PCy_3_ could be irradiated to produce similar results.

**Figure 6:**
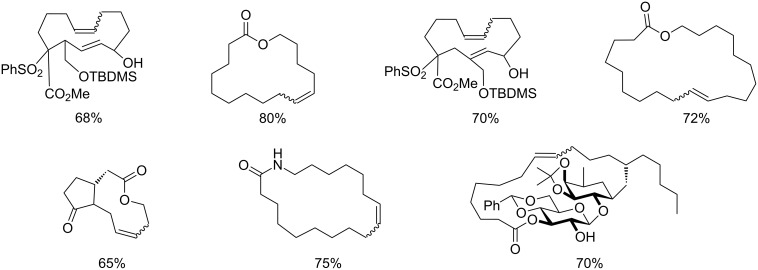
Some of the rings produced by photo-RCM.

Some of the photoinduced RCM products obtained by neon light irradiation of dimer **13** are highlighted in Figure 6. Perhaps the main benefit of this procedure lies in its simplicity since it only requires commercially available metal complexes and commonly used lighting equipment.

**Scheme 4:**
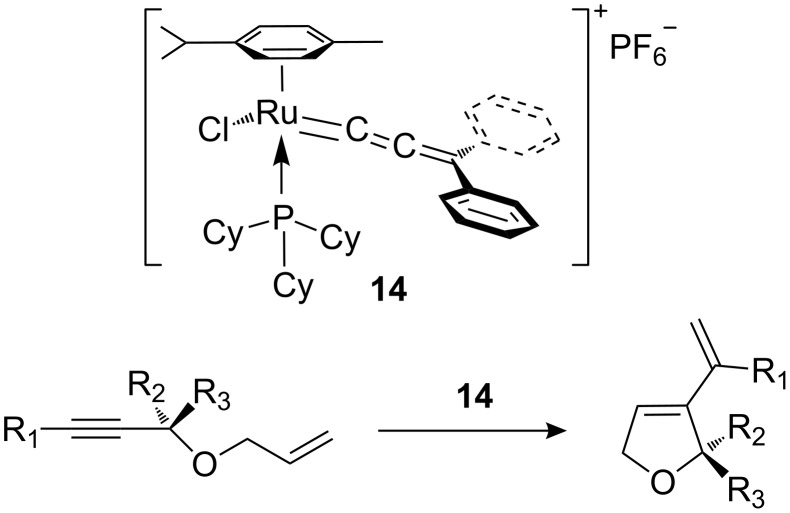
Photopromoted ene-yne RCM by cationic allenylidene ruthenium complex **14**.

An additional expansion to photopromoted RCM was described in 1998 by Dixneuf et al. [65], who used the cationic allenylidene ruthenium complex **14** (Scheme 4) for the ene-yne RCM of propargylic allyl ethers into 3-vinyl-2,5-dihydrofurans.

**Figure 7:**
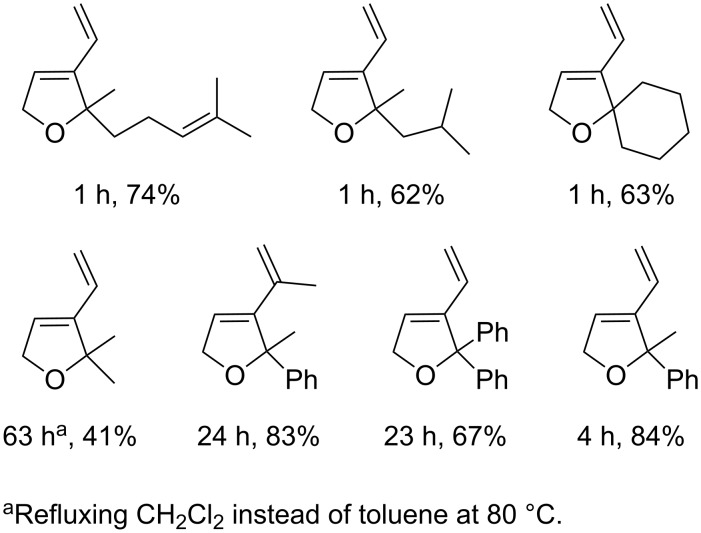
Dihydrofurans synthesised by photopromoted ene-yne RCM.

The best conditions reported for this reaction were the irradiation of a toluene solution of **14** and substrate with an Hg lamp at 300 nm for 30 min at room temperature, followed by heating at 80 °C until completion of the reaction. The reaction time, compared to the non-irradiated control experiment, was reduced six fold. The various dihydrofuran rings obtained by this method are shown in Figure 7 along with their respective yields and the required reaction times.

**Figure 8:**
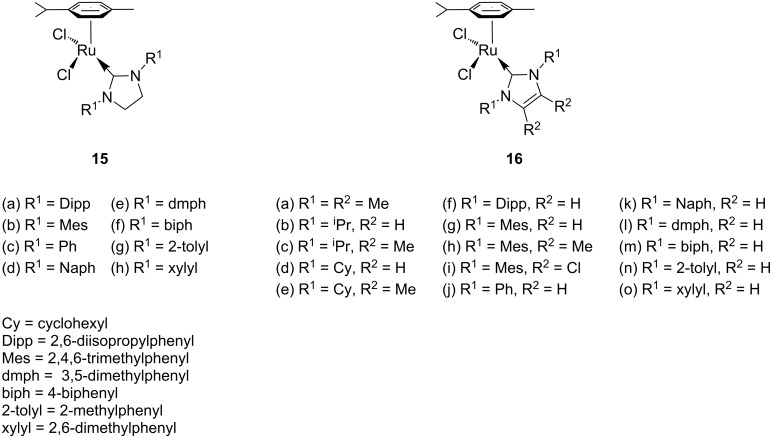
Ruthenium complexes with *p*-cymene and NHC ligands.

In line with the development of ruthenium benzylidene initiators [66–67], the phosphane ligand was replaced by an *N*-heterocyclic carbene (NHCs) in the photoactivated precatalysts. Accordingly, Noels et al. [68–69] synthesised a range of NHC substituted ruthenium cymene complexes (Figure 8) either by replacing the phosphane ligand in complex **12** by NHC ligands or via direct synthesis from **13**. Complexes **15** and **16** were tested as photoactivated ROMP catalysts. In all cases cyclooctene was used as a standard cyclic olefin monomer for the polymerisation studies.

Illuminating **15** or **16** with intense visible light, or even with regular laboratory lighting, revealed a dramatic improvement in their ROMP activity. Results of PROMP by complex **16g** (Mes ligand) and cyclooctene are summarised in Table 2. The large difference in polymer molecular weight between the dark and light reactions was not explained, even though it is slightly counterintuitive.

**Table 2:** Effect of light on the ROMP of cyclooctene using **16g** as catalyst.

Lighting conditions	Monomer conversion (%)	Isolated yield (%)	*M*_n_ (×10^3^ g)

Darkness	20	20	21
Normal^a^	93	84	625
Neon tube^b^	99	93	553
Light bulb^c^	>99	91	537

^a^normal lighting, a combination of daylight and fluorescent light; ^b^ordinary 40 W ‘cold white’ fluorescent tube, 10 cm away from the standard Pyrex reaction flask; ^c^250 W incandescent light bulb, 10 cm away from the standard Pyrex reaction flask.

In order to improve the understanding of the photoactivation mechanism, complex **16g** was irradiated by visible light both in the presence and absence of cyclooctene. NMR and UV spectra confirmed the release of *p*-cymene from the complex in the photochemical process; however, the active species and overall mechanism were not elucidated. Both saturated and unsaturated NHC ligands afforded similar results. However, blocking both *ortho* positions on the aromatic groups of the NHCs was crucial for the performance of the catalyst.

The ruthenium photoactivated catalytic systems described so far possessed noticeable ROMP activity at temperatures higher than room temperature even without being exposed to light; especially with the more reactive monomers such as norbornene and dicyclopentadiene. Buchmeiser argued that in order to integrate light activated precatalyst in practical applications, latency must be significant also at higher temperatures. Therefore, Buchmeiser et al. concentrated efforts towards the design of 'true' latent photoactivated ruthenium precatalysts for ROMP [70]. These precatalysts, similar to Noels’ complexes, included NHCs, and in some cases the *p*-cymene ligand was exchanged for phenylisonitrile and in others the chloride ligands were replaced by trifluroacetate. These transformations were expected to generate more stable, inert, precatalysts that would require external triggers to initiate the dissociation of the neutral ligand to produce the active species.

**Scheme 5:**
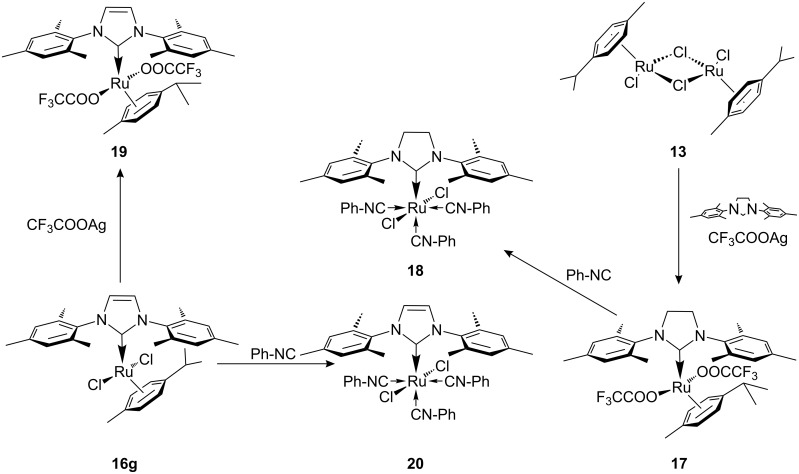
Ruthenium NHC complexes for PROMP containing *p*-cymene and trifluroacetate (**17**, **19**) or phenylisonitrile ligands (**18**, **20**).

Complexes **17**–**20** (Scheme 5) initiated ROMP of norborn-5-ene-2-ylmethanol **21** only at temperatures over 40 °C or by illumination at 172 nm. However, the reaction conditions required the removal of oxygen from the system, an encumbrance in practical applications. In addition, more reactive monomers, such as 2-norbornene, were thermally polymerised by the complexes even at room temperature.

**Figure 9:**
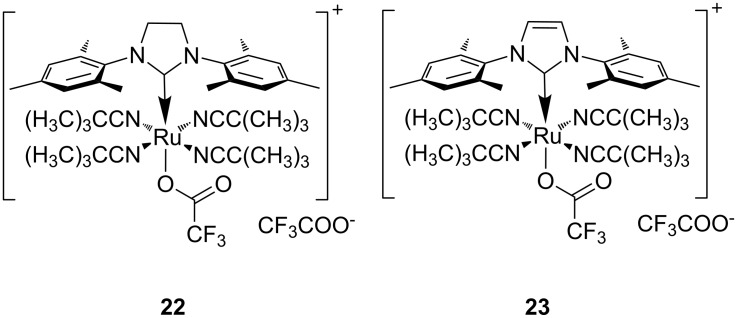
Photoactivated cationic ROMP precatalysts.

**Figure 10:**
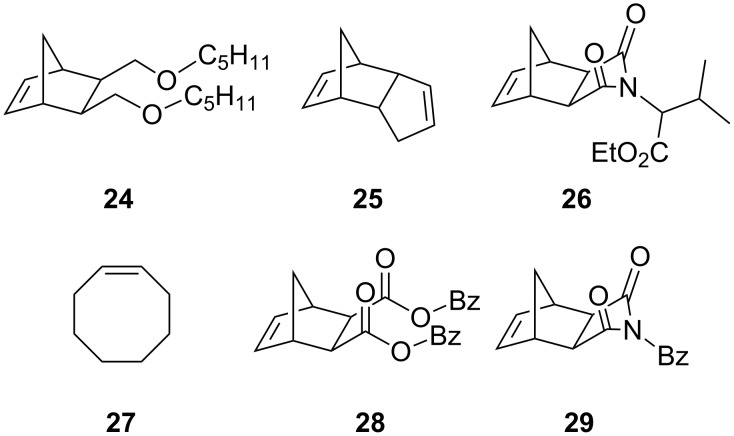
Different monomers for PROMP.

In 2008 Buchmeiser introduced the improved cationic latent phototriggered precatalysts **22** and **23** (Figure 9) [71]. These cationic species were inactive at higher temperatures (T < 45 °C) and did not thermally initiate the polymerisation of several ROMP monomers, including the highly reactive dicyclopentadiene **25** (DCPD) (Figure 10).

Irradiation at 308 nm of complex **22** or **23** in chloroform in the presence of the monomers resulted in polymerisations with 5–99% conversion yields (Table 3). On changing the light source to a 254 nm Hg lamp improved the yields to 70–99% (Table 3).

**Table 3:** Polymerisation results for monomers **24**–**29** by **22** and **23**^a^.

Catalyst	Monomer	Yield^b^		*M*_n_^d^
		308 nm	254 nm	

**22**	**24**	40^c^	95^c^	4.8 × 10^5^
**22**	**25**	82	99	—
**22**	**26**	69	85	2.1 × 10^5^
**22**	**27**	90	92	8.8 × 10^5^
**22**	**28**	<5^c^	90	2.6 × 10^5^
**22**	**29**	33^c^	99^c^	4.0 × 10^5^
**23**	**24**	41^c^	92^c^	—
**23**	**25**	>99	99	—
**23**	**26**	61	67	4.4 × 10^5^
**23**	**27**	91	90	8.8 × 10^5^
**23**	**28**	<5^c^	86	4.5 × 10^5^
**23**	**29**	21^c^	>99^c^	4.9 × 10^4^

^a^polymerisations were carried out in 5 mL CDCl_3_, monomer:initiator 200:1, 30 °C, 1 h; ^b^yield of isolated polymer; ^c^yield determined by ^1^H NMR; ^d^molecular weights measured for the polymers obtained with 254 nm irradiation.

**Scheme 6:**
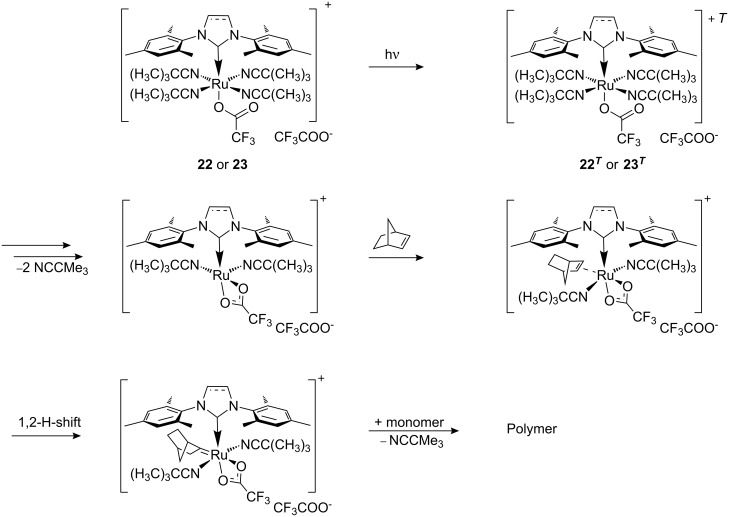
Proposed mechanism for photoinitiated polymerisation by **22** and **23**.

The proposed mechanism for the photoactivation of precatalysts **22** and **23** is displayed in Scheme 6.

**Figure 11:**
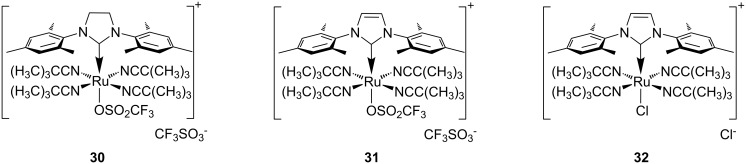
Light-induced cationic catalysts for ROMP.

Three additional new complexes for PROMP were recently published by Buchmeiser et al. [72] Although the complexes **30**–**32** (Figure 11) are not true latent precatalysts, only minor polymerisation occurred in the absence light (<5% at room temperature after 24 h). However, when the monomer–complex mixture was irradiated with a 254 nm UV source, polymerisation occurred with more than 60% conversion within 1 h in most cases.

**Figure 12:**
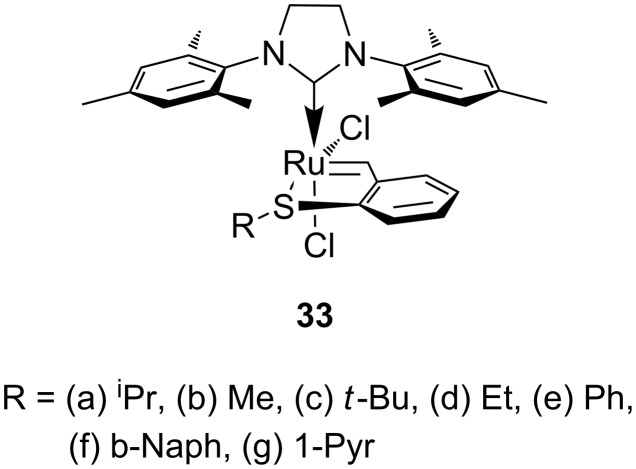
Sulfur chelated ruthenium benzylidene pre-catalysts for olefin metathesis.

A desirable enhancement in phototriggered catalysis is the generation of photoswitchable systems. Thus, a specific reaction may be turned on by one type of stimulus (heat, light), and turned off by another. We have recently developed latent sulfur chelated Hoveyda–Grubbs type complexes (Figure 12), as thermo-switchable catalysts for RCM and ROMP [73–77].

The thermal latency of the sulfur chelated complexes make them ideal candidates for photoswitchable applications; especially in light of the well documented photodissociation of ruthenium sulfur ligands.

Therefore, complexes **33a, e, f, g** were irradiated at 365 nm in the presence of RCM and ROMP substrates [78]. A summary of the results is presented in Table 4 and Table 5.

**Table 4:** Photo RCM of different substrates^a^.

Catalyst	Substrate	Product	Yield^b^
**33e** **33f**	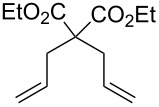	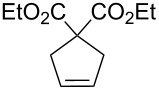	86% 77%
**33e** **33f**	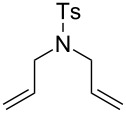	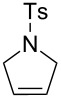	85% 78%
**33e** **33f**	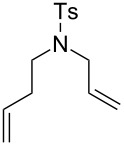	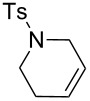	>99% 97%
**33e** **33f**	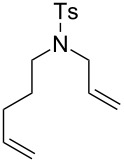	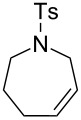	>99% 94%
**33e** **33f**	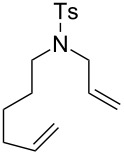	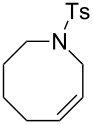	65%^c^ 54%^c^

^a^5 mol% catalyst, 0.1 M substrate in CH_2_Cl_2_ at 28 °C; 365 nm UV irradiation for 5 h; ^b^yields were determined by GC-MS after 24 h; no reaction observed without UV irradiation; ^c^includes isomerisation products.

**Table 5:** PROMP with sulfur chelated complexes^a^.

Monomer	Catalyst	Conversion^b^	PDI^c^	*M*_n_^c^

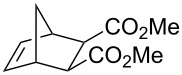	**33e** **33f**	40% 66%	1.5 1.5	2.5 × 10^5^ 2.5 × 10^5^
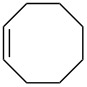	**33e** **33f**	96% >99%	1.3 1.2	1.1 × 10^5^ 1.3 × 10^5^
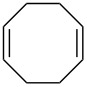	**33e** **33f**	86% 84%	1.4 1.5	5.0 × 10^4^ 7.0 × 10^4^

^a^monomer concentration 0.5 M in CH_2_Cl_2_; [monomer]/[cat] = 300; ^b^conversions were determined by GC-MS after 24 h with mesitylene as internal standard; ^c^*M*_n_ and PDI values were determined by GPC.

**Scheme 7:**
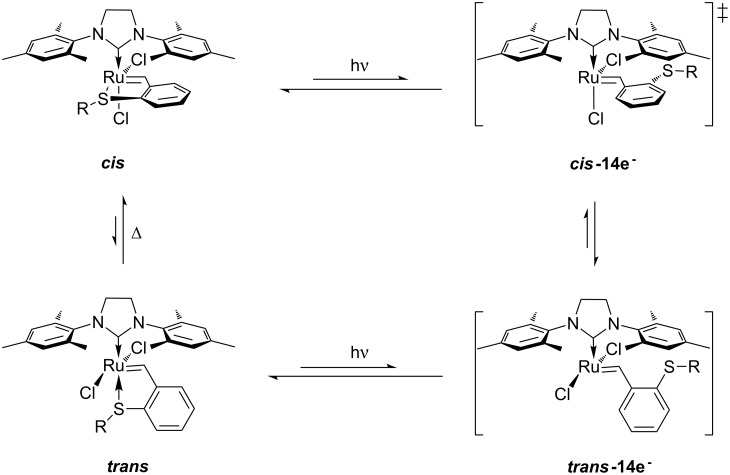
Proposed mechanism for the photoactivation of sulfur-chelated ruthenium benzylidene.

The discovery that light irradiation induces photoisomerisation of the *cis*-dichloro complexes led to the proposed mechanism shown in Scheme 7. Thus, photoactivation of sulfur-chelated ruthenium benzylidene complexes was found to depend on the generation of the active *trans*-dichloro isomer via 14-electron intermediates.

The design of a photoswitchable system was based on the fact that the latent isomer (*cis*-dichloro) was thermodynamically more stable than its active counterpart (*trans*-dichloro). Thus, illumination with UV light generates an active isomer, but a short heating period regenerates the inactive isomer. The switchable nature of the system was demonstrated by 15 min irradiation of a tetrachloroethane solution of diethyldiallyl malonate with 5 mol % of catalyst **33e** (activation), followed by 5 min heating at 80 °C (deactivation). Thus, whilst heating may be initially perceived as counterintuitive, it may be used to regenerate the latent species instead of activating it.

### Indirect metathesis photoactivation

An alternative approach for photoinitiated metathesis is indirect activation.

**Figure 13:**
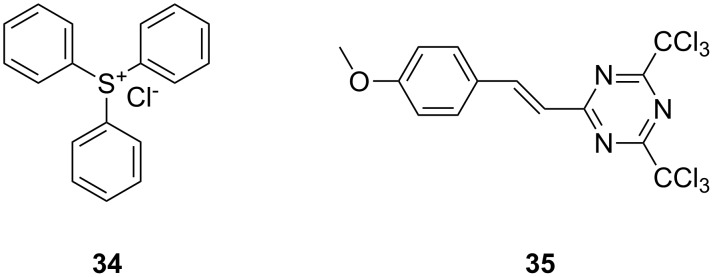
Photoacid generators for photoinduced metathesis.

**Scheme 8:**
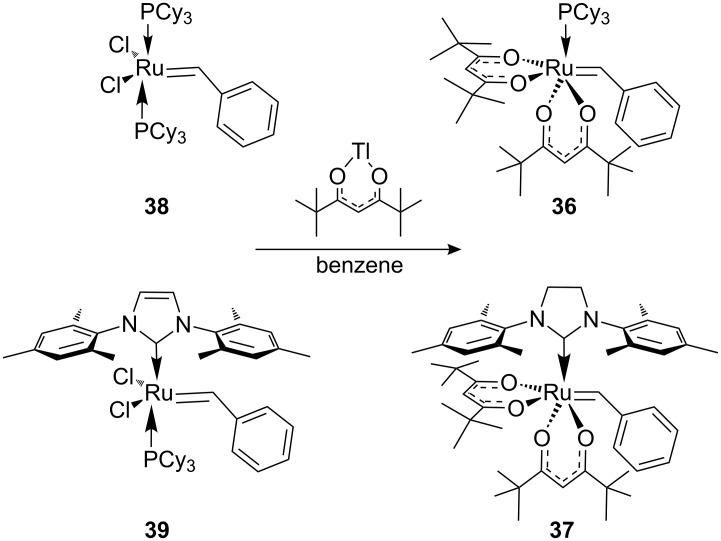
Synthesis of precatalysts **36** and **37**.

Grubbs et al. [79] demonstrated the use of photoacid generators (PAG) **34** and **35** (Figure 13) for the sub-300 nm UV activation of metathesis precatalysts **36** and **37** (Scheme 8). Thus, an acid sensitive olefin metathesis catalyst can be photoactivated by using a PAG in a tandem approach. Clearly, all other acids must be excluded from the reaction mixtures for the procedure to be effective.

Acid sensitive complexes **36** and **37** were synthesised starting from the Grubbs type catalysts **38** and **39** and thallium hexamethylacetylacetonate (Scheme 8). Several RCM substrates were closed as shown in Table 6 by the PAG induced metathesis. Complexes **36** or **37** and PAG **34** also polymerised typical ROMP monomers in excellent conversion (Table 7).

**Table 6:** RCM by PAG and precatalysts **36** and **37**^a^.

Catalyst	Substrate	Product	Yield

**36** **37**	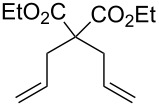	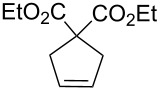	77% 83%
**36** **37**	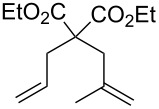	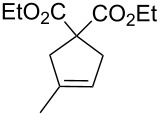	42% 88%
**36** **37**	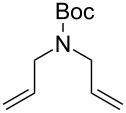	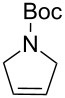	70% 93%
**36** **37**	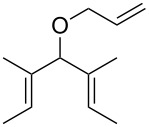	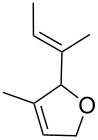	23% 62%

^a^5 mol % of **33/34**, 10 mol % of **31** and substrate in a quartz NMR tube in CD_2_Cl_2_ (0.1 M); ^b^isolated after column chromatography.

**Table 7:** ROMP by PAG and precatalysts **36** and **37**^a^.

Monomer	Cat.	Conversion^b^	PDI	*M*_n_^c^
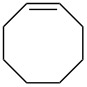	**36** **37**	>95% >95%	1.38 1.26	1.39 × 10^4^ 0.85 × 10^4^
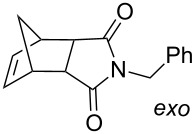	**36** **37**	>95% >95%	1.33 1.25	5.75 × 10^4^ 12.7 × 10^4^
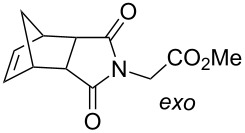	**36** **37**	>95% >95%	1.44 1.29	5.99 × 10^4^ 18.7 × 10^4^
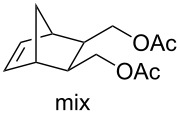	**36** **37**	>95% >95%	—^d^ —^d^	—^d^ —^d^

^a^**36, 37** (5 mol %), **34** (10 mol %) and monomer (0.1 M) in a quartz NMR tube with CD_2_Cl_2_; ^b^determined by ^1^H NMR spectroscopy; ^c^measured by MALLS-GPC; ^d^insolubility precluded GPC analysis.

**Scheme 9:**
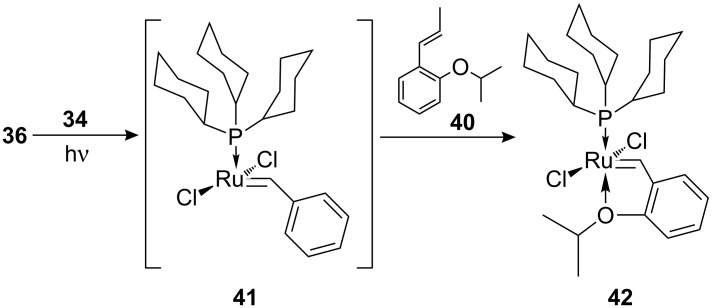
Trapping of proposed intermediate **41**.

A dramatic loss of activity was observed when the chloride anion of PAG **31** was replaced by the non-nucleophilic nonaflate ion, implying that the chloride plays a crucial role in the photoactivation of this system. A cunning trapping experiment using the isopropoxy aromatic derivative **40** (Scheme 9) provided a better understanding of the activation mechanism and led to the proposal of the well-known 14-electron complex **41** as the actual active species.

**Figure 14:**
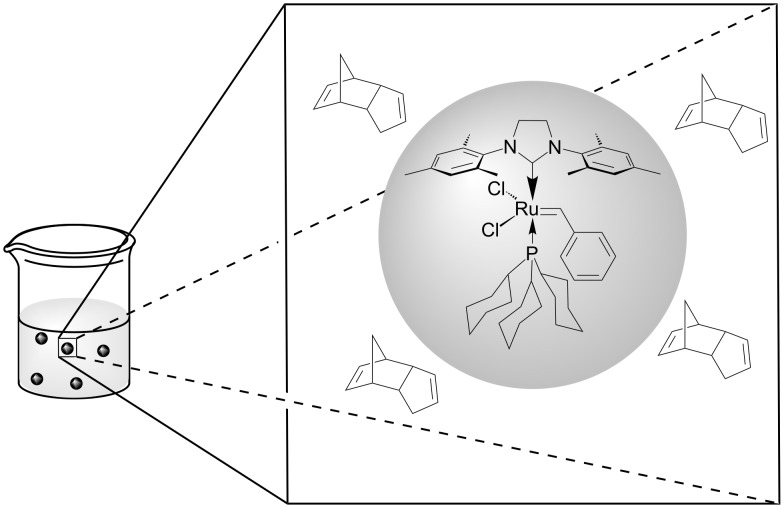
Encapsulated **39**, isolated from the monomer.

Another excellent example for nondirect activation of metathesis is the light-triggerable liquid-filled solid microcapsules (MCs) presented by Fréchet et al. in 2009 [80]. A solution of **39** in toluene inside macrocapsules (Figure 14) can stand for weeks in neat DCPD without any appreciable reaction. However, near-IR laser bursting of the MCs causes gelling due to polymerisation within min.

## Conclusion

Initially, the field of photoinduced olefin metathesis was mainly based on W(CO)_6_ chemistry. However, slowly over time functional group tolerant ruthenium applications emerged and these now dominate the field. Either by indirect or direct methods, the activation of ruthenium olefin metathesis catalysts may lead the way to novel applications in the areas of photolithography [81–82], roll-to-roll coating [69], 3D-printing, and self-healing [83] procedures in polymers. The use of photoswitchable catalysts may also have important safety advantages for industrial processes. As frequently seen in the successful field of olefin metathesis, the use of light to activate and direct these reactions certainly has a bright future ahead.
